# A Record of Personal Experiences

**Published:** 1918-10-05

**Authors:** 


### MEDICAL DISEASES OF THE WAR.*

### A RECORD OF PERSONAL EXPERIENCES.

A complete survey of the medical lessons taught by the war is a matter for the future. Though less in the public eye, and less dramatic in its character than that of the surgeon, the work of the physician has been highly necessary, and it has involved special responsibilities. The immediate responsibilities have of course been to the patients, but necessarily there has been a wider claim. Exceptional opportunities create exceptional obligations, and as the war has revealed new diseases, and has marked with a special character some of the older ones, and further, as patients in many of the groups have been numerous, the chance for careful and broadly-based clinical work has been great. In not a few instances this chance has been happily seized and has been used to good advantage. One of the best illustrations of this statement is the appearance of Dr. Hurst's book on "Medical Diseases of the War." The writer was well known prior to the war as an informed and competent physician; in the last four years he has enjoyed a varied experience both at home and in the East; and he has cultivated with success the art of observation and the faculty of lucid exposition. His book is a most engaging one and is rich in suggestions related to practical medicine. Necessarily it has a strongly personal note, but this is much to be desired, and constitutes, indeed, the special value of the contribution. Not that Dr. Hurst ignores the work of other observers. On the contrary, he is obviously well acquainted with this work. But special opportunities have come to him and he has set himself to see these with his own eyes and to appraise them with his own judgment. If there are individual points on which he fails to be convincing he never fails to interest his readers or to stir them to intellectual activity.

One of the clinical discoveries of the war is "Trench Fever." Pyrexia of unknown origin is a common enough experience in civil practice, and "influenza" during recent years has covered many such mysteries. In the Army, it seems, a similar custom was not uncommon, though the initials "P.U.O." also enjoyed official recognition and had the merit which properly belongs to a candid expression of ignorance. Comparatively early in the war such cases became numerous, and Major J. H. P. Graham in 1915 drew attention to a group in which two periods of pyrexia were separated by an interval of normal temperatures. This observation was confirmed from many quarters, and as the sufferers were confined to men who lived in or near the trenches the term "trench fever" became inevitable. At the same time it was observed that some of the orderlies in the hospitals to which the patients were removed became victims, and hence necessarily arose the suspicion that the disease was an infectious one and that it depended on some specific agent. The story of the investigations which were undertaken to discover this agent and to determine the medium or route through which infection was propagated is a most fascinating one, and can be read in Dr. Hurst's pages. The difficulties and the failures and the final success are good examples both of the conditions which attend many medical problems and of the fashion in which patience and ingenuity in conjunction with modern methods has led to confident knowledge. The conclusion now seems to be definitely certain—namely, that the cause of the disease is carried from man to man by lice. What exactly is the nature of the *contagium* carried by the lice has yet to be determined, and this aspect of the problem is at present being studied in France with the help of volunteers who allow themselves to be bitten by lice collected from men suffering from the disease. The whole record is an added chapter to the extraordinary development which has taught us in recent years how large is the part which insects are able to play in the spread of epidemic and endemic disease. Dr. Hurst's chapter on "Trench Fever" is a highly successful one.

Another chapter of considerable interest is the one which deals with "Beri-Beri." Though not often seen in this country except in seamen returning from the East, the disease has engaged widespread attention because of the observation that it occurred mainly among races whose staple diet is machine-polished rice, that is rice from which the husk and aleurone layer have been removed. On the other hand, the use of rice which has not been treated in this fashion does not produce the disease; further, such rice cures the disease if it has developed. Hence the necessary inference that the portions removed in "polishing" the rice contain something essential to the nutrition of the body and to the functions of the nervous and circulatory mechanisms. This "something" may not be capable of identification or of exact definition, but it makes all the difference between health and disease, and now it is appreciated as the representative of a wide class of bodies known as "vitamines," which have to be allowed to take their place, therefore, among the essentials of a healthy dietary. As Dr. Hurst points out, heat as used in the sterilisation of tinned foods destroys the vitamine which fresh foods contain, and hence a diet of this order unredeemed by fresh eggs, vegetables, or meat may cause the feeble circulation and peripheral neuritis which are the prominent features of beri-beri. As a matter of fact an outbreak of such a disease occurred among the British troops at Gallipoli. Yet Dr. Hurst doubts whether deficiency in diet was here an adequate explanation, and he draws attention to the frequency with which such symptoms followed an attack of jaun-

* *Medical Diseases of the War.* By Arthur F. Hurst, M.D., F.R.C.P. Second Edition. (London: Edward Arnold. 1918. Pp. 319 + vi. Price 12s. 6d. net.)

**Medical Diseases of the War——(*continued*).**

dice, as also to an earlier view (Hamilton Wright) of the relation of beri-beri to a toxic duodenitis. In any event he allows that the proper treatment is a diet rich in vitamines, and it is an illustration of the close relation of medicine to military efficiency to note that since the autumn of 1916 a preparation of yeast extract called "marmite," and rich in anti-beri-beri vitamines, has been added to the ration of the British troops in Mesopotamia. The result is seen in the disappearance of all severe cases, in the great reduction of the number of mild cases, and in the absence of any deaths. The moral cannot be too often repeated. It is that the discovery of the cause of a disease is the only sure way to effective treatment and, still more important, to successful prevention.

The earlier chapters of Dr. Hurst's book are concerned with nervous diseases and disorders, and he has evidently studied these conditions with special attention and enthusiasm. Broadly it may be said that he extends the area of functional disease into certain districts where hitherto a diagnosis of organic change would have been generally accepted; and he urges strongly the ability of suggestion, more or less vigorously applied, to remove promptly all symptoms which are essentially hysterical in nature. His experiences of functional disease, and especially of functional disease of the special senses, is a very wide and varied one, and his case-records of many of these are very forcible illustrations of the therapeutic practice which he advocates. A few instances of what may be called the popular view of the possibilities of severe nerve strain or shock may be noted. One of these is that of an officer whose hair turned white after he had been buried in a prone position for twenty-two hours, while a similar development occurred in a lad of seventeen years after exposure to a week's heavy bombardment. Again, in not a few cases after severe strain the hair was observed persistently to stand on end and occasionally this has continued after all other symptoms had passed away; not only the hair of the scalp has been affected, but also that of the trunk. The standing of the hair on end is, of course, a consequence of the overaction of the muscular fibres of the *arrectores pilorum*, but the exact mechanism of the sudden blanching of the hair remains a mystery. That it may result from organic injury to the central nervous system was shown by Gowers some years ago, and Dr. Hurst's case shows that, equally, functional disturbance may produce it. There are various well-known traditional examples of such development, but the evidence of these is not quite conclusive. It is therefore of special interest to have an instance guaranteed by competent medical observation.

Of the more serious and disabling war neuroses Dr. Hurst writes at length, and it is here probably that he will excite some measure of controversy. Not everyone will extend as widely as he does the significance of the term "hysteria," and there may be quarters where the claim made for the influence of suggestion, whether pathological or therapeutic, will meet with incredulity. Yet most certainly Dr. Hurst does not speak without chapter and verse, and it must be remembered that he is not the only physician who has described in recent years the ready and even immediate removal of the most extreme functional disturbances by treatment which pretends to be nothing more than pure suggestion even when this is associated with material agencies. Motor paralysis, tremors, contractures, such sensory symptoms as anæsthesia and sweating, as well as disorders of speech, sight, and hearing, all come within this category, and the incredulous must find some explanation of the actual records before their attitude can be justified. The facts assert themselves, and for them there must be some explanation. Dr. Hurst's records are those of a highly skilled observer, well equipped by training and experience to recognise organic changes when these really exist and little likely, therefore, to be deceived by appearances or to be led astray by an undisciplined enthusiasm. His observations go to show that the reign of hysteria is a more extensive and a more commanding one than has generally been supposed, and with this doctrine is associated the claim that by appropriate methods of treatment all such disturbances in the individual patient can rapidly and confidently be brought to an end.

In a short but very attractive chapter Dr. Hurst discusses the predisposing causes of war neuroses, and names as chief among these congenital nervousness, a previous nervous or mental breakdown, concussion and chronic alcoholism. Each man is endowed with a nervous system having particular qualities and tendencies, and these may in special directions be so extreme that neither training nor the will can control them. Thus some men are constitutionally brave, some constitutionally timid, and there are extremes of each type. There are men who actually do not know what fear is and who regard danger with a light and steady heart. Others, the majority, feel frightened and show their fear by such events as pallor, tremor, quickening of the pulse, etc., but conquering by an effort of the will this "absurdity of the flesh" they carry on with true courage. But what is possible in one direction is also possible in the other, and hence a certain number of men are physically hopeless as soldiers. They cannot by their wills command the physical and mental disturbances which the proximity of danger excites. They are "martial misfits," and while, in the ordinary conditions of life, they may show little or nothing out of the common, the battlefield makes them, sometimes greatly to their own surprise, nervous and incapable wrecks. The condition is independent of nationality and of race, though Professor L. R. Müller is quoted as responsible for the statement that in the Turkish Army—to which he was consulting physician—hysteria and other functional nervous disorders are unknown. In this respect as well as in many others Dr. Hurst's book provides material for the psychologist and even for the general reader as well as for the physician, and we have only to add that it is a most instructive, stimulating and engaging volume.

